# Forced migration, resettlement, and sport: Lessons from the Kabul-Edmonton soccer team

**DOI:** 10.1177/10126902241245769

**Published:** 2024-04-18

**Authors:** Jay Scherer, Ashraf Amiri, Dallas B Ansell, Paul Nya, Nancy LI Spencer, Nicholas L Holt

**Affiliations:** 3158 University of Alberta, Canada; 1851 University of Massachusetts, USA; 3158 University of Alberta, Canada; 3158 University of Alberta, Canada; 3158 University of Alberta, Canada; 2129 University of Calgary, Canada

**Keywords:** forced migration, resettlement, soccer, ethnicity, Afghanistan

## Abstract

Forced migration is one of the most pressing crises of our lifetime. Of the millions forced to migrate, many come to know the brutality of state-managed migration that habitually denies asylum seekers and places substantive restrictions on refugees who have been resettled. Sociologists of sport and leisure have examined the sporting experiences of refugees through an intersectional lens, foregrounding how displacement and resettlement are differently lived and negotiated across overlapping power structures and markers of gender, sexuality, ethnicity, religion, and legal status. Through a participatory and collective photovoice project, this article explores the experiences of an all-Afghan soccer team that played in a social, co-ed soccer league in the spring of 2022, just after they arrived in Edmonton, Alberta, Canada. In photovoice narratives and subsequent interviews, team members underlined many of the barriers they faced as they navigated the formal and informal rules and dominant norms of this seemingly inclusive sports landscape. In doing so, they revealed some of the limits of official discourses of Canadian multiculturism, which rarely accommodate more significant forms of difference, and which reproduce racial and ethnic hierarchies that powerfully discipline newcomers who are encouraged to embrace their precarious status as model minorities.

## Introduction

Forced migration is one of the most pressing crises of our lifetime. According to the United Nations High Commissioner for Refugees (2022), by the end of 2022, 108.4 million people were forcibly displaced due to persecution, conflict, violence, human rights violations, and climate change. Of the millions forced to migrate, many come to know the brutality of state-managed migration that habitually denies asylum seekers and places substantive restrictions on refugees who have been resettled. These practices have been further normalized in many Western countries in an era of emboldened white nationalism extremist movements and “deadly State and border policies” (De Martini Ugolotti and Caudwell, 2021: 2) that reproduce what Edward Said called *Orientalism* (1978). Orientalism remains a salient

Corporate institution for dealing with the Orient—dealing with it by making statements about it, authorizing views of it, describing it, by teaching it, settling it, ruling over it: in short, Orientalism as a Western style for dominating, restructuring and having authority over the Orient. (Said, 1978: 3)

The connections between forced migration and sport and physical culture, and the diverse experiences of refugees in these settings, cannot be disentangled from this broader political and historical context and the durability of this hegemonic system. Nor can they be disentangled from the geo-political forces and the colonial aspirations of empires that have dispossessed (and hence produced) “refugees” in the first place, rendering their lives and racialized bodies as less valuable, precarious, disposable, but also in need of saving.

There are over 2.6 million registered Afghan refugees in the world, and millions more have been internally displaced (United Nations High Commissioner for Refugees, 2021). These migration patterns have been amplified since the spring of 2021, when the United States (US) announced its withdrawal from Afghanistan, having occupied it for two decades. The rapid abandonment of Afghanistan emboldened the resurgent forces of the Taliban, which retook control of the country after capturing Kabul, Afghanistan's capital, in August 2021. Facing imminent threats to their lives, many thousands more—especially those from ethnic minority groups and those who had worked with coalition forces and with progressive non-governmental organizations (NGOs) in Kabul—abandoned their homes and fled.^1^

Nearly 6 months after the fall of Kabul, in January 2022, a group of 172 predominantly Hazara refugees arrived in Edmonton, Alberta, after the Government of Canada initiated new emergency resettlement programs for at least 40,000 Afghan nationals, including those with connections to Canada. The group that arrived in Edmonton consisted of educated, middle-class employees and families of Afghanistan Human Rights and Democracy Organization (AHRDO), a Kabul-based NGO dedicated to raising awareness about human rights issues, especially women's rights, and promoting peace and conflict resolution initiatives and transitional justice.^2^ This community of refugees shared a unique migration journey: after fleeing Kabul, they spent nearly 6 months together in Quetta and Islamabad, Pakistan, without documentation before being approved by the Department of Immigration, Refugees and Citizenship to travel to Canada. They are now rebuilding their lives in Edmonton under challenging circumstances as they seek to contribute to their communities, including participating in sport and physical culture. One of these practices was Kabul-Edmonton, an all-Afghan refugee soccer team, which played in a social, co-ed soccer league in the spring of 2022.

Sociologists of sport and leisure, especially in Australia, the UK, and Europe, have examined the diverse sporting experiences of refugees through an intersectional lens, foregrounding how displacement and resettlement are differently lived and negotiated across overlapping power structures and markers of gender, sexuality, ethnicity, religion, and legal status (De Martini Ugolotti and Caudwell, 2021; Jeanes et al., 2015; Smith et al., 2022, 2023b; Spaaij, 2015; Spaaij et al., 2019). Much of this research has moved beyond functionalist and policy-driven themes and questions around integration, well-being, and community cohesion, highlighting the recent assimilationist turn in political discourse and the resurgence of fascist/anti-migrant/Islamophobic political movements that are shaping the violence of migration management practices and the everyday experiences of refugees (De Martini Ugolotti and Caudwell, 2021).

Canada, which has experienced a rising tide of far-right extremism and violent Islamophobia since 2001, has seen a renewal of sociological research on the experiences of immigrants and newcomers in sport and physical culture (Barrick, 2023; Rich et al., 2015).^3^ Forced migration remains relatively under-explored, though, especially using participatory approaches that center the diverse voices of refugees as they navigate Western sports practices with their own histories of racism (Tink, 2022). Indeed, many of these sports are now being framed by sport organizations through benevolent discourses of inclusion and multiculturalism, often inferring that racism is a thing of the past.

But is Canadian settler society and its sports system as inclusive as these buzzwords suggest and as Canadians like to tell themselves they are? Who, exactly, is being included, and who is dictating the terms of inclusion in sport and in society (Donnelly and Coakley, 2002)? Have Canadian sport organizations addressed the well-known calls for the creation of just and equitable sports systems (Frisby and Ponic, 2013), and for the provision of genuinely democratized opportunities for newcomers, especially those who have experienced forced migration? Or are they simply upholding and reproducing the historical power of dominant groups and their own preferred ways of playing that produce uneven levels of inclusion for newcomers, as other studies have shown (Young and Block, 2023)? The provision of top-down opportunities framed around “developmental ideals” and discourses of inclusion can have problematic, if often unintended, consequences that amplify otherness for newcomers from refugee and forced migration backgrounds (De Martini Ugolotti and Caudwell, 2021).

In what follows, we engage these questions by detailing a participatory and collective photovoice project (Smith et al., 2023a, 2023b) that centers the voices of Kabul-Edmonton team members as they negotiated their first sporting experiences in a social, co-ed soccer league in the spring of 2022. Kabul-Edmonton's inaugural season was a challenging one; the team lost all but one of its games and almost imploded. But it did not implode, and the team's experiences contain important lessons about how this refugee community negotiated the complexities of a seemingly inclusive and social sports league at a crucial moment of their resettlement.

## Afghanistan: An often-interrupted history

Most Canadians know about Afghanistan, its people, and Islam through simplistic and problematic tropes circulated by Western news outlets and social media, especially following 9/11, when the Canadian Armed Forces invaded the country as part of the so-called “War on Terror” led by the US. These have been primarily Orientalist depictions of Afghanistan as a “perpetually failed state,” its history, diverse peoples, moral attitudes, landscapes, political structures, and cultures reduced and folded into a simplistic category that reproduces a common-sense binary of hierarchical power relations. Orientalism, as Said (1978) noted, is as much about defining the West and its cultural and moral superiority as it is about reducing the Middle East and its peoples to simplistic characters—terrorists and violent others, and/or victims and helpless bystanders—forever needing to be educated and saved by more rational and progressive people. These same Islamophobic tropes continue to shape the experiences of Afghan refugees, including those who now reside in countries that once invaded and occupied Afghanistan, like Canada, which maintained a military presence there until 2014.^4^

The history of Afghanistan and the broader region is, of course, far more nuanced, including eras fueled with optimism and hope when Afghans, not foreign empires, wrote their own histories and worked to reconcile the country's internal contradictions.^5^ Stability, modernity, and optimism characterized the “Golden Age” of the 1930s and the 1970s, for example, especially in Kabul, then the “Paris of Central Asia” (Bumiller, 2009). But Afghanistan's recent history has been “an often interrupted one” (Ansary, 2012), characterized by invasion, occupation, civil war, and violence. Ethnic and religious minority groups, especially the Hazaras, Aimaqs, Nuristanis, and various Pashtun tribes have been subjected to marginalization, violence, and genocide (Ansary, 2012; Hakimi, 2023; Jones, 2008).^6^ A rural–urban divide between the 70% of Afghans who live in rural areas and urban-dwellers, especially in the secular-modernist power center of Kabul, has contributed to brutal periods of civil war, culminating in the 1990s with the rise of the Taliban and its version of violent extremism.

For a brief interlude after 2001, parts of Afghanistan experienced relative stability, with improvements in education, the economy, infrastructure, and security. Thousands of Afghans who had been living in exile returned to Kabul, bringing with them progressive aspirations. The US and the international community poured money into the country, although much of it went to private firms and to foreign NGOs. Still, many NGOs, especially those staffed and run locally, “did truly vital work against great odds” (Ansary, 2012: 293). Many Afghans, especially those who came of age in these years, maintained a hope of playing a role in their country's reconstruction: they worked with coalition forces and with NGOs, advocating for peace, democracy, human rights, education, economic development, and the provision of sports opportunities, especially for girls and women.

The Taliban, however, gradually regained power in rural areas, capitalizing on the widespread sense of corruption and the lack of progress in building infrastructure by the expatriate Afghan technocrats in the central government; hostility to the secular urban culture of Kabul; changing gender relations; and outrage over the atrocities of US and coalition forces who had killed hundreds of thousands of civilians. The Taliban increased their attacks and suicide bombings at schools, sports facilities, and cultural events, slowly draining away “the aroma of hope” (Ansary, 2012: 318) that had been cultivated for the past decade.

In the spring of 2021, facing growing opposition to the presence of US soldiers in Afghanistan and widening domestic political divisions, President Biden declared the “War on Terror” over, naming a symbolic deadline of September 11 for the complete withdrawal of all US troops. In response, the Taliban expanded spring offensives and began marching to Kabul, taking control of the capital on August 15, 2021, prompting thousands to attempt to flee the city, including the group that eventually arrived in Edmonton in January 2022.

## Background to Kabul-Edmonton and methods

In early January 2022, the lead author was working on a community-based research project with Free Play (FP), a non-profit charity that provides sports programming for young immigrants and refugees in Edmonton, Alberta. FP had secured some ad hoc funding to run sport- and art-based activities for the children of the refugees from Afghanistan on weekday mornings, including free lunches. This funding was generously provided by Canadian Tire's Jumpstart program, and not the Department of Immigration, Refugees and Citizenship or by other levels of government. The community of refugees had been required to quarantine for 2 weeks in a hotel in downtown Edmonton due to COVID-19 restrictions, another period of suspended isolation that was retraumatizing for many. But for several weeks and in some cases several months, until their families found homes in Edmonton, Afghan children were picked up by buses at their hotel and accompanied by FP to an indoor soccer facility.

The lead author supported FP staff, including Carlos, who was responsible for the organization's programming for the Afghan children; Carlos was a crucial gatekeeper for the research. Crucially, many FP staff, including Carlos, are migrants themselves and intuitively connected with the children, even with a not-insignificant language barrier that required creative solutions. Other FP staff had women's studies backgrounds and were attuned to the gender dynamics of sports programming and the challenges facing girls and women in Afghanistan. One morning, when FP staff were picking up children at the hotel, Shuja—with the support and encouragement of others—asked Carlos whether opportunities existed for the adults, and they exchanged phone numbers to continue the conversation. As one of the older group members, and one of the few who was fluent in English, Shuja held a gendered position of responsibility and was afforded customary status and respect from younger group members.

Shuja had been a refugee before, having fled Afghanistan to Pakistan with thousands of others following the Soviet invasion in 1979. Soccer was a crucial, life-sustaining activity in those refugee camps, and a key site of sociality to connect with host communities and with individuals from different religious and cultural backgrounds. Fast forward to 2022: Shuja and many others knew that all the refugees—the men and, crucially, the women—needed these opportunities in Edmonton and to be with their children in this early period of rebuilding their lives together. One of the few things that Shuja took as he fled Afghanistan for the second time in 2021 with his wife and three young children was his pair of indoor soccer shoes (Shuja, personal communication, February 22, 2022). In contrast to some depictions of refugees as dependent, desperate, and perpetually grateful for the benevolence of host communities and non-refugees, these constituted actions of agency and purpose to recreate a life of hope, health, sociality, and well-being amidst life-changing circumstances.

The Afghan adults began coming to the soccer center with their children; the men and the women alternated every week, and sometimes played together, cheering when they first arrived at the soccer fields. For Ibrahim, one of the older refugees in his early 60s, after months of confinement and uncertainty, arriving at the soccer fields “felt like freedom” (Ibrahim, personal communication, June 22, 2023). The lead author and FP staff played, too, but with “Kabul rules,” a version of futsal, and with most communication in Dari. Contrary to dominant understandings of modern sport as universal, or as a one-directional gateway between “developed and developing worlds” (Mashreghi et al., 2021: 158), games were played on the refugees’ terms, minimizing the reproduction of othering in this context. There was no *lingua franca* on the field; the community of refugees was learning (and wanted to learn) English, and they encouraged FP staff and the lead author, in turn, to commit to learning as much Dari as possible.

This was also the first time that the lead author met the second author, who held considerable status in the group: he was fluent in English, served as the translator for all, and had completed his Master's degree in conflict resolution in the US before returning to Kabul. During these early conversations, many refugees reflected on how challenging the resettlement process continued to be, and how important it would be to have opportunities to be physically active in the weeks and months ahead, *on their terms*. After several months of suspended time, they were now running out of it, facing pressing deadlines for significant issues: finding homes, registering in English language courses, meeting with various non-profit organizations, setting up bank accounts, enrolling their children in schools, all while grieving and dealing with the trauma of what they endured. The first and second authors agreed that these were stories that needed to be told, including the role of sport and physical activity during a challenging resettlement. They initiated discussions about how this could be done as ethically and as collaboratively as possible, eventually focusing on photovoice as a unique and appropriate method to support social justice-focused research, one that can democratize the research process and shift power relations and control to participants (Hayhurst et al., 2016; McHugh et al., 2013). Indeed, many of the refugees were already chronicling their journeys from Kabul and were well aware of the power of representation.

Over the next 2 months, most families found homes in apartment buildings in West Edmonton, a suburban, working- and middle-class community. Many Afghan children and youth continued participating in FP's after-school programming and camps, and some young men and a few women expressed their own aspirations to play competitively in the spring and summer. Others simply wanted to play soccer recreationally in the early stages of their resettlement as an extension of their bonds of friendship and solidarity. As a migrant from Colombia and a soccer player and coach himself, Carlos was familiar with the near-insurmountable obstacles that often preclude these aspirations for newcomers—transportation, equipment, exorbitant fees, negotiating dominant sports cultural norms and systems (Young and Block, 2023)—but he had the cultural capital and connections to help aspirant players surmount them.

Carlos noted that a co-ed, all-Afghan team could be registered in the spring to play in a social soccer league to provide a casual introduction to the local sports landscape. And, as Carlos underlined, because this was a co-ed league, FP might fund the team given the organization's commitment to gender equity and to the provision of democratized sport opportunities. On the other hand, significant uncertainties remained: Was there enough interest in the refugee community to field a co-ed team? Would there be enough women to play? How would it work, and who would manage it?

The second author used existing networks on WhatsApp to communicate in Dari with prospective players, while Carlos and the lead author lobbied the CEO of FP to cover the costs of registration fees, equipment, and transportation; FP secured funding from Canadian Tire's Jumpstart program. Within days, this ad hoc process, with the support of non-profit organizations and the private sector, led to the formation of a co-ed soccer team. The team's name, Kabul-Edmonton, a name of enormous significance for the community, was chosen by the second author and was enthusiastically endorsed by the prospective players over WhatsApp. Team organizers were conscious of the types of power relations that often structure top-down, paternalistic sporting experiences for newcomers. Collectively, it was agreed that the second author would be the team's manager, spokesperson, and coach; the lead author and Carlos, in turn, would play supporting roles as assistant coaches/managers.

This was also when the first and second authors realized the genuine possibility of a community-based participatory research (CBPR) project that explored the diverse experiences of Kabul-Edmonton team members as they navigated their first co-ed recreational soccer season. CBPR presumes knowledge generation to be a collaborative process of inquiry, one that necessitates democratized engagement with co-producers as equal partners “to harness and enhance the strengths within the community to address concerns and inequities” (Mayan, 2023: 129). Together, the lead and second authors committed to ensuring the co-creation of equitable, ethical, and adequately resourced partnerships that included those with lived experiences in making important decisions throughout all phases of the research process—from the development of research questions to dissemination in ways that shared power and control (McSweeney et al., 2022; Robinson et al., 2019; Spaaij et al., 2018). As the start of the Kabul-Edmonton season approached, the lead and second authors began to discuss research ideas and potential methods with team members, eventually settling on photovoice: everyone had access to smartphones. Six men (Arsalan, Mahmood, Mohammad, Delawar, Ekhlas, and Eskander) and two women (Fatima and Khadija) expressed interest in co-developing the project and agreed to take pictures of their experiences throughout the season.^7^

In the discussions that followed, ideas and broad research questions quickly took shape as the team began to practice and play together. Kabul-Edmonton team members aspired to explore their experiences as a community of Afghan refugees in this inaugural co-ed soccer season, and how they negotiated the gendered and racialized power relations that shape, and are shaped by, a seemingly social and inclusive Western soccer league. To fully understand their complex negotiations of the normative standards of a co-ed social sport league in Canada, team members wanted to outline the intersecting inequalities that had shaped their varied sporting experiences playing sport in Afghanistan and then as refugees in Pakistan, especially along the lines of gender.

They also wanted to underline that their experiences and aspirations to play soccer in Edmonton were not just about integration or development; they were looking forward to having fun, meeting other soccer players, and reclaiming some form of control over their bodies and lives, while nurturing their nascent bonds of friendship and solidarity as a community of Afghan refugees. Finally, team members also talked about the importance of generating lessons for organizations like FP, Jumpstart, and various levels of government from the perspectives of the refugees themselves to illuminate the different levels of inclusion and exclusion that shaped their experiences. These discussions extended a focus to education and political action as a form of activist research, with the aim of shining a critical spotlight on Western institutions, an unsurprising development given the activist backgrounds of many in the group.

Those involved in the photovoice project discussed extensively the labor of this research to ensure that co-producers did not feel overwhelmed or exploited (Smith et al., 2023a, 2023b). Knowing that money was in short supply, it was agreed that co-producers would be reimbursed $25 per week to provide photos and an accompanying narrative (*n*  =  30) for each of the final 4 weeks of the soccer season, and an additional $25 for a final post-season interview/review of photovoice submissions with each co-producer (*n*  =  8; six men and two women). Semi-structured interviews were also conducted with five other players on Kabul-Edmonton (Iman, Ismail, Shuja, Kamran, and Ibrahim, all men) who did not have the time to participate in the broader photovoice project. The average length of interviews was 1.5 h. Funds were distributed by the second author weekly in cash and with no strings attached. The second author also collated and translated the weekly photovoice submissions, and arranged subsequent interviews, which were conducted by the lead and second authors. The second author's capacity as a translator, and his status as a trusted, well-known, and respected member of this particular Afghan refugee community, was crucial in facilitating open, far-reaching, and impactful conversations.

Co-producers were encouraged to be creative: to take pictures of their experiences on and off the pitch, and to write about them in ways that were meaningful to them. Photovoice can be a powerful, visual method, especially for capturing representations of lived experiences and the emotions that accompany them (McSweeney et al., 2023). A picture can be worth a thousand words, but it can also have unintended consequences: To prevent any risk to participants, and to their families who still live in Afghanistan under the Taliban, we haven’t included pictures in the analysis.

The first and second authors reviewed all interviews, pictures, and photovoice narratives in an initial round of open coding to become familiar with all that had been shared. This was, at times, heavy emotional labor, especially for the second author. Together, the first and second authors then engaged in an iterative process of thematic coding in relation to the broad research questions and ideas that co-producers had generated and engaged with during the soccer season. Following the conclusion of these initial rounds of coding, the first and second authors met with co-producers to collectively discuss and analyze the main themes that had emerged. Co-producers took this opportunity to reflect more deeply on their experiences; indeed, many of the themes they had underlined in their photovoice narrative submissions and in subsequent interviews had only become more salient and affective as time had passed.^8^

## Football habitus: Historical levels of inclusion and exclusion

One of the main themes in the literature on the sporting experiences of refugees is that many bring with them a well-cultivated football habitus and varying levels of physical and cultural capital, which can support them through challenging resettlement processes (Dukic et al., 2017). These often-gendered attributes and embodied dispositions, from a surplus and strengths perspective, supported many of the refugees from Afghanistan, especially through their inclusion in the Kabul-Edmonton football team, but in unequal ways.

All of the young men who took part in the Kabul-Edmonton photovoice project and in subsequent interviews recounted how recreational and competitive football and futsal had played significant roles in their lives in Afghanistan, bringing them immense joy and happiness. They grew up in the post-Taliban era, when playing opportunities in Afghanistan expanded in rural and urban communities, especially as new infrastructure was built. A generation of boys and young men, especially those from an expansive middle class in Kabul, embraced playing soccer and grew up at a time when the Internet provided them with unprecedented access to global popular culture, opening the possibility of being part of a transnational community of football fans and consumers. Many of the young men in the refugee group, in other words, shared similar elective affinities and embodied gendered dispositions, typified by Eskander's remarks:

I have been playing soccer/football for many years; I have played in Kabul, Iran, and now I play in Canada. I played three times a week in Kabul, and it was part of my weekly activities. We were always happy in Kabul, and playing football was joyful because we were with our families. (Eskander, photovoice submission)

As cultural and gender relations changed, many young men in this generation embraced new opportunities to play football recreationally with girls and women—their sisters, cousins, and friends—although they had yet to share mixed competitive sport experiences.

The older men on Kabul-Edmonton and in the community of refugees, like Ibrahim and Shuja, lived through decades of conflicts that devastated rural and urban infrastructure, but they still found ways to play soccer, even when it was dangerous to do so, especially under regimes that exhibited contradictory approaches to sport. In the 1990s, under the Taliban, for example, sports were officially discouraged, even for men, as a potential site of gambling (Ansary, 2012). Still, men's soccer games and leagues were permitted and were immensely popular, drawing crowds of thousands in urban centers like Kabul. The Taliban used those events as opportunities for public spectacles of torture and execution, including the stoning of women at halftime (Ansary, 2012).

Unsurprisingly, the women in the group had different sporting experiences and possessed different levels of physical and cultural capital. Some of the younger girls and women who grew up in the post-Taliban era, particularly those in urban centers like Kabul, embraced newfound opportunities to play soccer and to be physically active, cultivating their own football habitus in ways that an older generation of Afghan women could only dream of. Still, only one or two of the young women in the group had played competitive soccer in Afghanistan; a handful of others had played recreationally. Fatima, who had played competitive soccer, reflected in her photovoice narrative:

I started playing soccer when I was 11 years old. I was on a girls’ team named Adalat. Back then Afghanistan really was Afghanistan [post-Taliban, 2001]. But playing soccer or any other sport was too hard in Afghanistan. People said that sport was not for girls and girls had to stay at home and learn housework. But I continued to play.

Then we were told not to go out for unnecessary reasons because everyone thought the Taliban would come back. My Mom did not want me to go out a day before the Taliban came to Kabul, but I did go out and attended my last exercise. When the Taliban retook Kabul, I thought I would never play soccer again. (Fatima, photovoice submission)

In the current era of Taliban control, women's sport has been all-but-extinguished, as has access to secondary and university education. Girls and women are banned from competing in sport and exercising in gyms, while the Taliban intimidates and harasses those who once played, often scaring them from even exercising in private (The Associated Press, 2023). These stories underline the fact that there is no standard, singular narrative into which every refugee fits neatly, especially with respect to their sporting experiences and backgrounds that intersect with other aspects of their identities—age, gender, ethnicity, class, legal status among others (De Martini Ugolotti and Caudwell, 2021)—to produce varying levels of capital and different embodied histories that would play out on the soccer pitches of Edmonton with Kabul-Edmonton, and, earlier, on the pitches of Quetta, Pakistan.

## Cultivating connectedness in Quetta: Gendered soccer experiences

Following their varied and challenging migrations into Pakistan, the refugees assembled in a distinct part of Quetta called Hazara town, a religious and cultural traditional community that contains a large Afghan diaspora. They lived in a communal wedding hall for just under a month, men, women, and children under one roof, grappling with significant amounts of “waiting time”; they had no way of knowing how long they would be in Pakistan or where they would eventually go.

Their living arrangements and mobility in Quetta were challenging for several reasons. Due to the area's traditional, religious customs, men and women had to be separated at most times; girls and women were required to wear hijab at all times. Local residents informally policed these customs, and the group of refugees continually felt the weight of this surveillance: they were vulnerable to being reported to Pakistani authorities and deported if they were found to have violated these rules. Money was also running low; most had left Kabul quickly and with few possessions. They were undocumented in a foreign country and faced great uncertainty; these conditions frayed their physical well-being and autonomy.

For many, moreover, this was their first time meeting fellow refugees. Soccer, as it turned out, played a key role in cultivating new bonds of friendship, attachment, and solidarity, helping to restore their humanity and their agency in ways that transgressed the rigid spatial limitations and confines of their provisional shelter. But only for the men. Due to the area's religious customs and cultural expectations, it was impossible for girls and women to play soccer with the men or to play on their own in an unfamiliar community at such a vulnerable moment. Likewise, domestic and childcare responsibilities and a traditional gendered division of labor set powerful limits and pressures on the availability of “free time” in this setting. The men, on the other hand, enjoyed greater privileges and mobility, and they eventually sought out opportunities to rent a nearby soccer pitch, even risking apprehension by Pakistani police in the process. As Ekhlas noted in his photovoice narrative,

Few of us knew each other when we arrived in Quetta, Pakistan. The first step to getting to know friends, and I should say the best way to make friends, is sports. And in those hard days it became much harder without football and sport. We saw a lot of hardships in Quetta; even the children were depressed. Together, we took the children with us to play football, even though they did not understand everything, and that helped get rid of the depression. (Ekhlas, photovoice submission)

These brief experiences of joy and fun reinfused their connections to their own bodily and mental health at a time of significant uncertainties around food, sleep, and well-being, but they were distinctly gendered experiences. In cultivating spaces of care and mutual aid through soccer in Pakistan, and in cultivating physical capital and friendships, many of the men in the group also developed aspirations to continue to play together wherever they resettled. However, after traveling to Islamabad, the capital of Pakistan, in early November, where they faced even more daunting security restrictions as undocumented refugees, not even the men dared to venture outside to play soccer. Finally, after 2 months of uncertainty and stress in Islamabad, as they waited for the Government of Canada to approve their resettlement applications, the group traveled to Edmonton in January 2022, which brings us to their experiences of inclusion and exclusion on the co-ed Kabul-Edmonton soccer team that spring.

## We decided to leave the team

The social league is designed to provide inclusive, co-ed sports opportunities. But the league remains structured according to dominant Western values of competition and domination and preferred ways of playing that have historically privileged the physicality of many men. And, while co-ed leagues and new rules around inclusion are designed to transform these gendered hierarchies and competitive structures, they often have unintended consequences. The co-ed league, for example, requires all teams to maintain a minimum of three women on the field (out of eight players). Again, this rule was designed to create an inclusive, social sporting environment with different guiding principles; people of all gender identity or expression are welcome to play in the league. But the rule is also contradictory: in a competitive sport setting, it signals to women that they are inferior, and requiring only three women on the field (instead of four or five), encourages a hyper-competitive environment in which some men become dominant.

For Kabul-Edmonton, the rule was challenging for several reasons. First, out of the community of 172 refugees, only a few women had any experience with “sport” whatsoever. Religious and cultural customs and traditions also created insurmountable barriers for some. Out of the handful of women who had played soccer in Afghanistan and who had initially expressed an interest in playing in Edmonton, many were unwilling to share the field with men on a co-ed team, underlining the barriers for Muslim girls and women in participating in mixed-gender sports environments (Kay, 2006; Walseth and Fasting, 2003; Young and Block, 2023). Similar religious traditions and cultural dynamics also made some of the men hesitant to play on a mixed team. Girls’ and women's participation in physical activities, especially in public spaces, and engaging in team sports with men, has long been a contested issue within Afghan families, and remains so to this day, including for communities in exile.

Of the 12 women who initially registered to play for Kabul-Edmonton, only two remained by the start of the season. In a message to team managers, one of the women underlined the intersecting, multidimensional dynamics at play that set limits on the ability of women migrants and refugees to participate in sport (Hurley, 2019):

The first reason is that almost everyone is busy now; they don’t have time. The second reason is that they don’t have practice, so they feel that they are not useful and can’t play. And, some people have the problem that they can’t play with men; they want to play football, but they say only women. (Anonymous, personal communication, May 2, 2022)

Behind the scenes, many of the men were having similar conversations, including around traditional gender roles. While many had played competitive sport in Afghanistan, no one had ever participated in a co-ed team before. As Mohammad explained in his photovoice narrative submission,

…[lots of] Afghanistan is traditional [where] no one likes girls and women to play soccer or any sports because there are a lot of restrictions; only men can do everything, but not women. But in Kabul, it was a little bit better than anywhere else in Afghanistan. Girls and women could play soccer, but many families didn’t allow them to. (Mohammad, Photovoice submission)

Kabul-Edmonton could not assemble a full complement of women players and forfeited its first game, playing most games with less than eight players on the pitch. Opposing teams, as it turned out, were hesitant to “lend” any of its women players to play on an all-refugee team, especially if it could potentially cost them points. And even when three women were available to play on Kabul-Edmonton, there were no subs who could come on to allow them to catch their breath. There were always, on the other hand, subs for the men. Recognizing these challenges, early in the season, Kabul-Edmonton asked the league if it could field fewer women and more men given the team's unique background. The team explained that Kabul-Edmonton was not trying to circumvent a rule to gain a competitive advantage: they simply wanted to play. The league denied their request (Anonymous, personal communication, May 15, 2022): Other teams struggled to find women players, so why should Kabul-Edmonton receive favorable treatment?

The inflexibility of the league's rules and its rationalist structure exacerbated tensions, almost resulting in Kabul-Edmonton's withdrawal. In this instance, a rule that had been designed in the spirit of inclusion and sociality had unintended consequences for newcomers, and for racialized women in particular: they were “the problem” (again), and they were well aware of it. Kabul-Edmonton collectively discussed recruiting women players from outside the Afghan refugee community to help “make up the numbers.” While the men, who were in the majority, supported this decision, Fatima and Khadija were outraged, and with only a few games left in the regular season, they walked away from the team. As Fatima recounted in her photovoice narrative:

We told them not to bring women from the outside and that we will work on it ourselves. …[but] they said they were going to … and we got a little angry that they didn’t pay attention or didn’t care about what we said. And when we lost the next game, they put the blame on the girls. We decided to leave the team. For the next few weeks, we didn’t play. But we knew they needed more women. So we decided to go and play with them. We went to the field, we talked and we solved the problem we had, and we started playing again.

In search of reconciliation, Kabul-Edmonton team members had several collective and individual discussions and eventually found a constructive solution. To maintain the team's initial commitment—that Kabul-Edmonton would field an all-Afghan team of men and women—it was agreed that other women *from the Afghan diaspora* would be invited to play. Team members demonstrated great capabilities in addressing this internal challenge, but it required a series of hard discussions and considerable emotional labor. Before the final playoff game of the season, with the support of Fatima and Khadija, Kabul-Edmonton invited three women from the Afghan diaspora community to join the team. Coordinating these invitations was not straightforward: it was a last-minute solution that took considerable time and energy, and it was successful only because of newly formed relationships with other members of the Afghan diaspora.

The league's aspiration to provide inclusive, social sports opportunities from a Western perspective, created a hyper-competitive, gendered environment that marginalized the players of Kabul-Edmonton, especially the women. As noted by Dukic et al. (2017: 103), the inclusion frameworks of many sports organizations are often best “defined as the absence of exclusive policy, as if to say ‘we’re an inclusive club, anyone is welcome to join and play.’” But these frameworks are often little more than window dressing that reproduces an assimilationist agenda and, hence, the status quo. Kabul-Edmonton team members were always welcome to participate in the league, but their involvement was restricted to playing under conditions not of their choosing and without any genuine influence in shaping the broader context of their experiences. Kabul-Edmonton could have simply withdrawn from the league as an act of refusal, but this would have marked this team of refugees as failures, and it would have been a devastating end to their first sporting experiences in Canada. In facing this double-edged sword, team members decided to make adjustments to keep playing, albeit under conditions that reinforced their otherness.

## One of the most upsetting games; One of the happiest days

As a team of racialized refugees, Kabul-Edmonton team members anticipated they would have an outsider status in the league before their first game of the season, even in a sport as diverse as soccer. While the team's name—Kabul-Edmonton—was a matter of pride and reverence, it also marked the team as radically different from others that had embraced names like “Ball Busters FC” and “Up Your Arsenal.” As refugees, moreover, team members were conscious of the Orientalist baggage they embodied: they looked and sounded different; and they had never played this style of co-ed soccer. They were also increasingly aware that they were living under heightened conditions of surveillance that underlined their otherness during their resettlement. As Ismail noted, “The government is watching you, Catholic Social Services is watching you, what do you, what do you do? What courses do you take? They have an eye on you with everything” (Ismail, personal communication, August 25, 2022).

Some other teams and players, including those from racialized backgrounds, had little patience as Kabul-Edmonton adjusted to the official and unwritten rules of the league, occasionally subjecting them to sarcastic comments like “you’re not from here, are you?” and other normalized practices of trash talking that took aim at their backgrounds and ethnicity. The experiences of overt and subtle forms of racism often leave racialized refugees questioning if they will ever participate in soccer again (Baker-Lewton et al., 2017), a sentiment that was echoed by some members of Kabul-Edmonton.

Kabul-Edmonton endured other challenges on the pitch: the soccer league is self-officiating, but matches are facilitated by Games Coordinators who often act as referees. Some of the Games Coordinators recognized that the refugees were new to the league (and to the country) and cut them some slack on certain rulings, but others surveilled Kabul-Edmonton in irrational ways that reinforced the team's status as outsiders. Certainly, these dynamics always have a subjective element, but it is indisputable that most Kabul-Edmonton team members could not easily communicate with Games Coordinators (or with opposing players) to dispute calls in ways that other teams could, including those comprised of racialized players who spoke English fluently. Kabul-Edmonton's subordinate position in this racial and ethnic hierarchy proved disadvantageous on several occasions, and, as the season progressed, team members became increasingly angry with their treatment by some Games Coordinators and opposing players. These tensions erupted during the team's first playoff game.

Kabul-Edmonton started this game with only two women players. And despite enjoying this advantage, a few men on the opposing team were finishing tackles in ways that contravened the rules and codes of the social league, while boasting and showboating after goals that came in quick succession. They were also successfully intimidating the Games Coordinator, himself an immigrant, who repeatedly penalized and scolded Kabul-Edmonton team members, ignoring similar infractions by the opposing team. Tempers erupted early in the first half. After being repeatedly subjected to offensive remarks by opposing players, some of the men on Kabul-Edmonton gestured angrily at the Games Coordinator, yelling in Dari at several men on the other team. At halftime, a meeting was held between the two team captains to determine if the game should be called off. Cooler heads prevailed, but Kabul-Edmonton lost by a score of 7–3, and team members were despondent and incensed. As Mahmood reflected in his photovoice narrative, “It was one of the most upsetting games. The referee was taking sides. When we shouted, the referee started behaving a little bit differently by the end of the game” (Mahmood, photovoice submission).

The comments that Kabul-Edmonton was subjected to by opposing players were devastating to team members. As Iman explained in a follow-up interview, “During that game … they were telling us ‘You guys are nothing. You came from a country that is full of war’. I heard that many times during that game. That was the saddest game” (Iman, personal communication, July 22, 2023). Shuja, who had earlier in his life experienced a more welcoming and inclusive soccer culture as a refugee in Pakistan, underlined his disappointment and the devastating impact of his experiences:

I wanted to interact with these people, to meet them, to know them better. That was our first experience in Edmonton. We didn’t expect to win, we just wanted to experience the new environment. When you hear such things, it sometimes kills you from inside. (Shuja, personal communication, July 22, 2023)

The behavior of the opposing players and the Games coordinator on that day—the direct or indirect unfair treatment of, or unfavorable attitudes toward, Kabul-Edmonton players based on their appearance, language, ethnic background, and names—exemplifies the act of othering in ways that reflect Islamophobic attitudes and Orientalism. It also reveals the racial and ethnic hierarchies and the “invisibility bargain” (Pugh, 2021) beneath the veneer of official discourses of Canadian multiculturalism: the unwritten, but strongly enforced, sets of expectations placed upon newcomers to conform to hegemonic structural conditions and dominant norms and standards; to be docile, and to minimize or hide forms of radical difference, including language, religion, and other cultural customs. In other words, to be perpetually grateful, model minorities: English-speaking, hard-working, disciplined, and, eventually, middle-class/bourgeois, whose differences lie only on the surface, and who must “perform complete allegiance to the norms of the nation” (Kalman-Lamb, 2013: 243), including the norms of its sports cultures. In pushing back on the field that day—by gesturing angrily and shouting in Dari and by challenging the status quo of white privilege—Kabul-Edmonton team members broke the invisibility bargain, and at a significant emotional cost. It is one thing to lose a game, but it is quite another to be othered so dramatically as a team of Muslim refugees whose membership in the imagined, multicultural, national community was always tenuous at best. The damage had been done.

Or had it? The following week, after meeting and collectively deciding to play the team's final playoff game, Kabul-Edmonton recruited additional women players from the Afghan diaspora, earning a 5-2 victory that sparked an eruption of joy. Two weeks later, the team and family members and friends celebrated the end of the season at a park in central Edmonton. In light of this seemingly fairy-tale ending, what are the main lessons that can be drawn from the experiences of team members of Kabul-Edmonton in this co-ed social sports league?

As a community of refugees who mostly came of age during a certain era in Afghanistan's history, the men and women of Kabul-Edmonton had vastly different sporting experiences in Kabul, and also in Edmonton. Kabul-Edmonton was expected to fully comply with the league's stringent rules around gender, almost resulting in the team's collapse. While the league supported sociality and inclusion, these understandings were top-down constructs, drawn from Western understandings that don’t always welcome, or expand to accommodate, the diversity of other cultural traditions and experiences. Kabul-Edmonton was expected to integrate into the league in a one-way manner, and with little assistance, even in a sports culture as diverse as soccer. When, despite expending significant effort and emotional labor to be “included,” Kabul-Edmonton players were publicly subjected to racist invective, they broke the invisibility bargain, amplifying their otherness in profoundly alienating ways.

Throughout the season, no social bridges were made between Kabul-Edmonton players and those from other teams. In a group discussion, not a single player from Kabul-Edmonton could even recall a gesture of greeting or support from another team. As Shuja noted, these experiences made a stark contrast to the significant and ongoing support the community of refugees received from schools, from their neighbors in West Edmonton, and from FP and other social service providers:

The other teams would probably Google the name Kabul-Edmonton, and then search for Kabul, where it is and what's going on there. I wish they would have just come to us and communicated in a different way, to welcome us. It would have been lovely if they told us, “hey, even if you guys lose, you are the winners for us. You are the champions because we know what's going on in Afghanistan. People are being tortured, people are being killed, you are going through trauma, you're going through a lot of mental health and psychological challenges.” So that's what we hoped the other teams and the sports setting in Canada would do. But the reality was, unfortunately, they did not. (Shuja, personal communication, July 22, 2023)

Many of the team's experiences, however, especially the final game, were also joyous and fun, allowing them to get a better sense of Edmonton and its sports landscape; Kabul-Edmonton was also a steppingstone for several of the men and women who now play on men's and women's soccer teams in the Edmonton and District Soccer Association. Still, as a unique, co-ed soccer team of Afghan refugees, Kabul-Edmonton's experiences on the pitch underlined their precarious status and existence in this sports culture. Kabul-Edmonton did not return to the field in the following season.

## Conclusion

Through a unique and collaborative photovoice project that centers the voices of Afghan refugees with forced migration backgrounds, we have explored the experiences of Kabul-Edmonton team members who participated in a co-ed, social soccer league in the spring of 2022, just after they arrived in Edmonton, Alberta. In photovoice narratives and subsequent interviews, team members underlined many of the barriers and difficulties they faced as they navigated the formal and informal rules and dominant norms of this seemingly inclusive sports landscape. In doing so, they revealed some of the limits of official discourses of multiculturalism, which rarely accommodate more significant forms of difference, and which reproduce racial and ethnic hierarchies that powerfully discipline newcomers, encouraging them to embrace their precarious status as model minorities.

Significant work remains to further democratize and transform dominant sports cultures in Canada, especially to support refugees who have been driven from their homes and countries as a result of violent extremism. And while refugees from forced migration backgrounds share many similar challenges with racialized immigrants/economic migrants as they attempt to navigate the sport system—limited financial resources and time, family and ethno-racial group influences, language and cultural considerations, limited social networks, challenges with the sports bureaucracy, and racism—there are also differences in their experiences. Extensive funding, in particular, is needed to support those who have fled conflict and have arrived in Canada with minimal financial resources and without documentation; indeed, these issues can pose unique challenges for refugees as they navigate complex and costly sports systems. Extensive funding is also needed for trauma-informed translators, social workers, and coaches who are sensitive to the experiences of refugees from conflict-driven, forced migration backgrounds, and their ongoing navigation of trauma, including worrying about the loved ones and friends they’ve left behind, and whose lives remain endangered.^9^ There is also a need for more informal opportunities, including other therapeutic arts-based activities, that allow refugees from forced migration backgrounds to shape their experiences during resettlement and to heal on their terms.

Sport organizations across Canada, however, are also initiating important efforts to recruit and welcome refugees and immigrants into many sports. The implementation of these measures, though, is uneven, and constrained by the broader political and institutional structure within which sport has historically been governed in Canada. Certainly, at the high-performance level (funded by the Government of Canada), some national sport organizations have initiated anti-racist policies and training for staff and coaches (Barrick, 2023). At the community sport level, which often operates outside of the formal sport system and still relies on volunteers for participation-based activity, there are less resources for anti-racist policies or training; very few community and recreational sport organizations track, or account for, racist incidents in ways suggested by sport-related bodies like Anti-Racism and Sport, developed by Immigration Partnership Winnipeg (IPW).

In pursuit of these aspirations, all levels of government need to provide funding to meaningfully democratize sport in ways that move beyond assimilatory and integrationist agendas that do little to challenge hegemonic conditions. Indeed, if the Government of Canada, which has committed to ramping up its immigration targets while continuing to resettle refugees from Afghanistan, is going to offload these responsibilities to non-profit organizations, those organizations need more consistent and defined resources to assist with this crucial work.^10^ Otherwise, official discourses of multiculturalism, including photo-ops like the one taken by Sean Fraser, the former Minister of Immigration, Refugees and Citizenship who played soccer with the Afghan refugees in Edmonton in February 2022, will ring hollow.

Finally, while the non-profit industrial complex can offer significant support to the sports-migration resettlement field, its reliance on powerful institutions/funders and top-down program designs can perpetuate, even if unintentionally, a wide range of inequalities, including prioritizing assimilation instead of refugee-driven agency and empowerment. Within their constraints, non-profit organizations need to center migrant voices, advocate for systemic/fundamental change with community members and activists, and encourage culturally sensitive sports programs that challenge the status quo with the broader aim of promoting democratization and social change well beyond the playing field.

## Notes

1. These include a special immigration program for Afghan national and their families who assisted the Government of Canada; a humanitarian program to resettle vulnerable Afghans; a pathway to permanent residence for extended family members of foreign interpreters; and a program to sponsor Afghan refugees without refugee status from the UNHCR or a foreign state. For more information, see https://www.canada.ca/en/immigration-refugees-citizenship/services/refugees/afghanistan/special-measures.html.

2. Among the group who arrived in Edmonton were the second author, his wife, their three children, and the second author's younger brother and sister.

3. This recent work builds on a sizeable body of research on the sporting experiences of immigrants in Canada (e.g., Campbell et al., 2016; Curtin et al., 2018; Frisby, 2011; Stack and Iwasaki, 2009; Taylor and Doherty, 2005; Tirone et al., 2010).

4. 159 Canadian soldiers died on missions in Afghanistan; another 22 died in non-combat circumstances.

5. During this era, much of the country experienced remarkable growth and democratic reform, especially in education, and rapidly changing gender relations. Kabul has been home to diverse communities and holds immense political, economic, educational, cultural, and historical significance. The city represents modernism in the country, and most of the political aspirations and forces that have sought to democratize the country have been cultivated there.

6. The “Pashtuns have dominated national politics in Afghanistan since the foundation of the Afghan state by Ahmad Shah Durrani in 1747” (Barfield, 2014: 96). This dominance has remained largely unchallenged, with the exception of two short interruptions in the 1920s and 1990s when Tajik leaders served as emir and president of Afghanistan (Barfield, 2011).

7. Pseudonyms have been used for all participants.

8. Ethical approval was received for this project by Research Ethics Board 1 at the University of Alberta, Pro00110294.

9. For example, in October 2022, a suicide bombing at the Kaaj Higher Educational Center in Kabul killed over 50 people, mostly young girls and women. The bombing, which targeted Hazaras, had a pronounced impact on the group of Afghan refugees who had arrived in Edmonton in January; some lost friends in the attack, and others had family members who were seriously injured. A subsequent march was organized in Edmonton to denounce the ongoing genocide of Hazaras, culminating in a rally at the Alberta Legislature Building.

10. In 2023, the Government of Canada announced that it had met its stated goal of resettling at least 40,000 Afghans, and that this resettlement work will continue. Many Afghans who fled to Pakistan and who have applied for resettlement in Canada, including those who assisted the Canadian government, continue to face enormous uncertainty and threats of abuse, detention, and deportation back to Afghanistan, where they would face torture and other persecution (Dickson, 2023). Afghan women and girls face even greater barriers to resettlement: destination countries, like Canada, have prioritized men who assisted with military efforts (ibid).
